# A Review of Tunable Wavelength Selectivity of Metamaterials in Near-Field and Far-Field Radiative Thermal Transport

**DOI:** 10.3390/ma11050862

**Published:** 2018-05-22

**Authors:** Yanpei Tian, Alok Ghanekar, Matt Ricci, Mikhail Hyde, Otto Gregory, Yi Zheng

**Affiliations:** 1 Department of Mechanical, Industrial and Systems Engineering, University of Rhode Island, Kingston, RI 02881, USA; yanpei_tian@my.uri.edu (Y.T.); alokg@my.uri.edu (A.G.); mhyde64@my.uri.edu (M.H.); 2 Department of Chemical Engineering, University of Rhode Island, Kingston, RI 02881, USA; matthew_ricci@my.uri.edu (M.R.); gregory@egr.uri.edu (O.G.)

**Keywords:** wavelength selectivity, near-field, far-field, radiative heat transfer, metamaterials

## Abstract

Radiative thermal transport of metamaterials has begun to play a significant role in thermal science and has great engineering applications. When the key features of structures become comparable to the thermal wavelength at a particular temperature, a narrowband or wideband of wavelengths can be created or shifted in both the emission and reflection spectrum of nanoscale metamaterials. Due to the near-field effect, the phenomena of radiative wavelength selectivity become significant. These effects show strong promise for applications in thermophotovoltaic energy harvesting, nanoscale biosensing, and increased energy efficiency through radiative cooling in the near future. This review paper summarizes the recent progress and outlook of both near-field and far-field radiative heat transfer, different design structures of metamaterials, applications of unique thermal and optical properties, and focuses especially on exploration of the tunable radiative wavelength selectivity of nano-metamaterials.

## 1. Introduction

Thermal radiation of most naturally occurring materials is predicted by the Stefan-Boltzmann law and by Planck’s law as the distance of two emitting/absorbing bodies is much larger compared to the characteristic wavelength of thermal radiation whose physical mechanism is interpreted by Wien’s displacement law [1]. As the geometric parameters of two emitting/absorbing bodies approach the characteristic wavelength of thermal radiation, the interference of evanescent waves and photon tunneling contributes to the tremendous increase in magnitude of the radiative heat transfer between two objects [2]. Thus, the artificial metamaterials show an extensive emission spectrum, while emissive properties of nanostructured materials are remarkably different from those of natural bulk materials. Thermal metamaterials show great potential in the development of selective thermal emitters or absorbers which play a major role in the progression of solar cells and thermophotovoltaics (TPVs) and infrared thermal sensing applications [3,4,5,6,7,8,9], thermal diodes [10,11], radiation cooling [12,13,14,15], thermal rectification [16,17], biosensors, and chemical sensors [18,19]. Nanomaterials/nanostructures have become the topic of many articles, which focus on energy conversion and thermal management, such as one or two dimensional metal-dielectric periodic structures [20], multi-layered structures of thin films [21], nanoparticle-embedded thin films [22,23], hyperbolic metamaterials [24,25] and phase changing metamaterials [26]. All of these structures give the desired narrow emission spectrum, angular spectrum, or both because the structures produce some interactions with electromagnetic waves. For photonic crystals, the scale of the structure is usually on the order of the wavelength of thermal emission. However, for plasmonic and metamaterial structures, the characteristic wavelength is much smaller compared to the wavelength of thermal radiation [21].

Radiation heat flux is magnified by many orders of magnitude because of the coupling of surface waves which is defined as the near-field thermal radiation. This takes place when the gap between two emitting/absorbing objects is at the same order of magnitude of thermal wavelength [27], many research groups have experimentally confirmed that radiative heat transfer between planar surfaces separated by a vacuum gap smaller than the thermal wavelength exceeds the blackbody limit due to tunnelling of evanescent modes [28,29,30,31]. Further investigations found that the enhancement of radiation heat flux can occur at a specific wavelength, not over the broad range of emission spectrum due to the coupling of surface plasmon polaritons (SPPs) or surface phonon polaritons (SPhPs) across the two surfaces.

Rapid developments in theoretical, computational, and experimental investigation as well as in engineering applications have taken place in recent years due to advances in computational abilities and nanofabrication technology. This article intends to provide a brief summary of recent progress in theory, calculation, metamaterials of various structure, and different engineering applications.

The structure of the paper is as follows. In Section 2, we mainly discuss different structures of metamaterials. In Section 3, we present the recent progress of several engineering applications. Subsequently, we provide some viewpoints regarding future opportunities and feasible engineering applications that will emerge in this field.

## 2. Diverse Structures of Metamaterials

There is a large number of metamaterial structures that have been discussed in many published articles, such as nanoparticles embedded multi-layered structures, periodical grating structures, hyperbolic metamaterials, multi-layered structures and phase change metamaterials. Though materials and geometries can vary, these devices all serve to shift optical properties to enable useful function.

### 2.1. Nanoparticles Embedded Multi-Layered Structures

**Figure 1:**
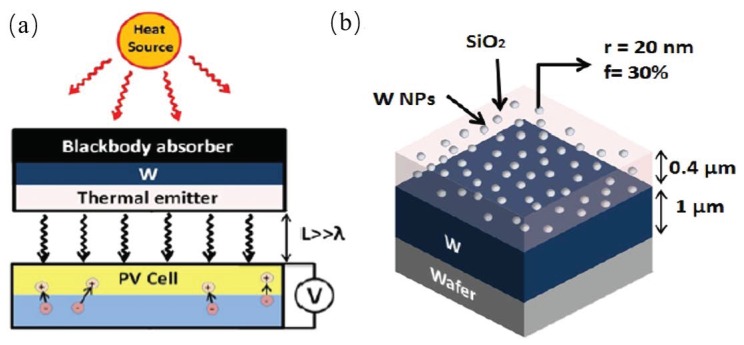
(**a**) Schematic of a typical TPV system with a thermal emitter/absorber and a PV cell. (**b**) A proposed design of thermal emitter consists of 0.4 μm thick SiO${}_{2}$ layer on the top of 1 μm thick W layer deposited on the substrate. SiO${}_{2}$ layer is doped with W nanoparticles of 20 nm radius with a volume fraction of 30%.

A mie-resonance metamaterial design of thermal emitter, which has a structure of nanoparticles-embedded thin film was proposed by Ghanekar et al. This structure can be used in the thermophotovoltaic system (TPVs), as shown in Figure 1a. The emitter is composed of a thin film of SiO${}_{2}$ on the top of a tungsten layer with randomly distributed tungsten nanoparticles embedded and deposited on the substrate, the thickness of the SiO${}_{2}$ and tungsten layer is 400 nm and 1 μm, respectively, as shown in Figure 1b. The embedded tungsten nanoparticles alter the refractive index of the SiO${}_{2}$ film, which brings about the desired emission properties in the wavelength range of 0.4 μm to 2 μm matches well with the External Quantum Efficiency (EQE) GaSb and InGaAs based photovoltaics. Maxwell-Garnett-Mie theory was applied to calculate the effective dielectric properties and the co-sputtering method was introduced in the nano-structures fabrication [23].

### 2.2. Periodical Grating Structures

#### 2.2.1. 1-D Grating Structures

**Figure 2:**
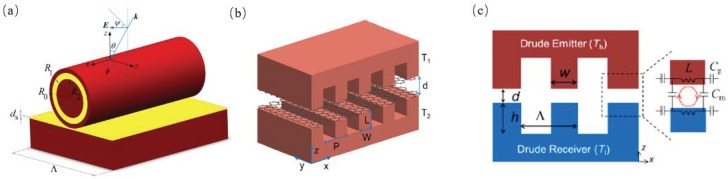
(**a**) Schematic of the unit cell of the 1D periodic Ag/ SiO${}_{2}$/Ag core/shell coaxial cylinders lying horizontally on a planar substrate [32]. (**b**) Schematic of radiative heat transfer between graphene-covered silica gratings separated by a vacuum gap of distance *d*, where *P*, *W*, and *L* are the grating period, width, and depth, respectively [33]. (**c**) Schematic of the radiative transfer between two symmetric, perfectly aligned metallic gratings with parameters of period (Λ = 2 μm), depth (*h* = 1 μm), and ridge width (*w* = 1 μm) [34].

A structure of Ag/SiO${}_{2}$/Ag coaxial cylinders on top of a SiO${}_{2}$ layer with a silver substrate, as shown in Figure 2a, was demonstrated by Zhao et al. They used the Comsol Multiphysics software based on the finite element method and explored the absorptivity of that structure with different silver core radii, spacer shell thickness and one core/four shells coaxial cylinders [32]. Liu et al. conducted an investigation into the near-field radiative heat transfer between two graphene-covered SiO${}_{2}$ hinges on exact scattering theory, as shown in Figure 2b, and found that the radiative heat flux was enhanced by more than one order of magnitude between two SiO${}_{2}$ grating structures [33]. Yang et al. theoretically demonstrated that the exciting magnetic polaritons can be enhanced by the near-field radiative heat transfer within the 1D periodic grating structure divided by vacuum gaps, the proposed structure is shown in Figure 2c [34].

**Figure 3:**
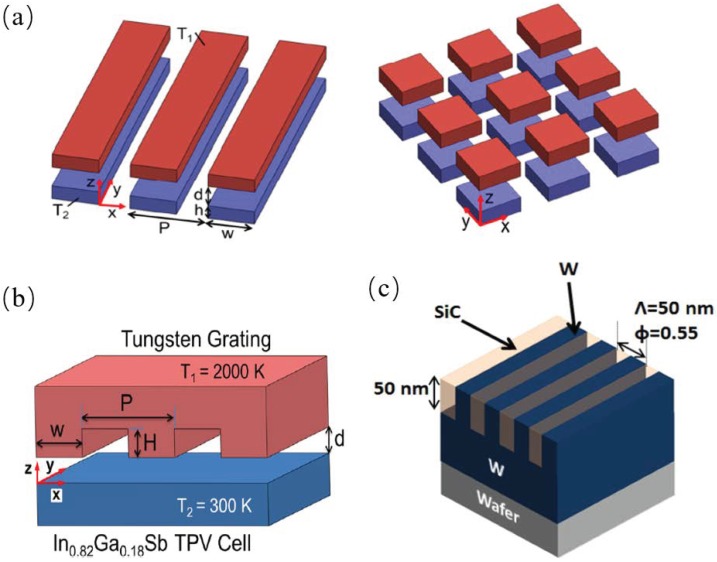
(**a**) Schematic of near-field radiation between 1D and 2D metasurfaces separated by a vacuum gap of *d* with temperatures of T${}_{1}$ and T${}_{2}$, respectively. Note that *h* is the thickness, *P* the period, and *W* the width of the 1D or 2D patterns [2]. (**b**) Schematic of the NFTPV device showing the coordinate axes, vacuum gap spacing *d*, and the geometric grating parameters: period *P*, height *H*, and ridge width *W*. The temperatures of the emitter at T${}_{1}$ and receiver at T${}_{2}$ are specified [35]. (**c**) An example of a thermal emitter based on 1-D grating structure of SiC and W on the top of W base. The grating thickness and period *L* = 50 nm, filling ratio *f* = 0.55 [23].

Based on the scattering theory formulations, Liu et al. proposed one 1D and 2D metasurfaces, as shown in Figure 3a that can enhance the radiative heat transfer to more than one order of magnitude when the thickness of the metasurfaces were at a certain range [2]. Watjen et al. proposed a near-field thermophotovoltaic system which consisted of a high temperature tungsten grating structure which generated photons to a room-temperature In${}_{0.18}$Ga${}_{0.82}$Sb PV cell across a vacuum gap at several tens of nanometers, as shown in Figure 3b. They used the RCWA together with scattering theory to calculate the radiative heat transfer between the thermal emitter and the PV cell. Ghanekar et al. investigated the wavelength selective properties of the 1D grating structure, as shown in Figure 3c, this structure has high emissivity in the region which the GaSb or InGaAs based PV cell has high external quantum efficiency.

#### 2.2.2. 2-D Grating Structures

**Figure 4:**
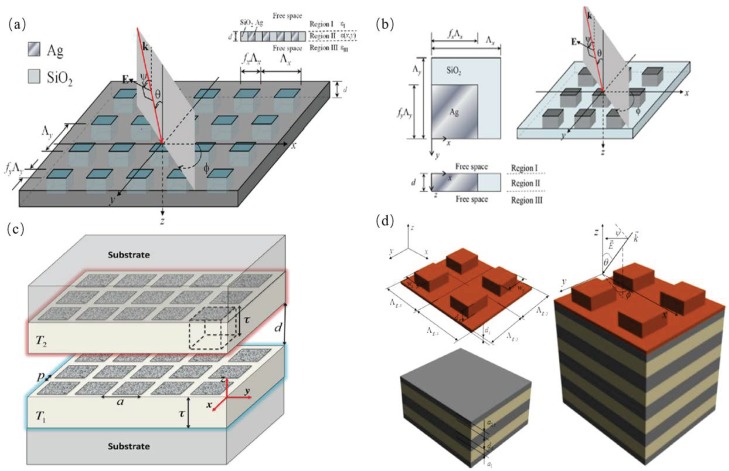
(**a**) Schematic drawings of proposed 2-D (*x* and *y* directions) periodic Ag inlaid with SiO${}_{2}$ nanostructures [36]. (**b**) Periodic Ag nano-pillars embedded in a SiO${}_{2}$ film at oblique incidence. Magnified top and side views of a structure period are also shown [37]. (**c**) Schematics of two doped-Si metasurfaces made of 2D periodic arrays of square holes placed on semi-infinite planar substrates and held at temperatures T${}_{1}$ and T${}_{2}$. The key parameters are shown: lattice parameter (*a*), distance between holes (*p*), gap size (*d*), and metasurface thickness (τ) [38]. (**d**) Schematic view of the 2D grating/PC structure [39].

Huang et al. proposed and optimized a new energy-saving glass with silver blocks embedded within SiO${}_{2}$ thin film nano-structures using numerical methods, as shown in Figure 4a. Based on the rigorous coupled wave analysis algorithm (RCWA), they demonstrated that the proposed structure has the desired wavelength-selective reflectance (*R*) and transmittance (*T*) [36]. The energy-saving glass has a high *T* and low *R* in the visible range, while the low *T* and high *R* in the near-infrared which saves energy for building lighting and diminishes the infrared radiation and then decreases the cost for air-conditioning [36]. Figure 4b shows a periodically-isolated pattern nanostructure consisting of silver nano-pillars embedded in the SiO${}_{2}$ blocks which were designed by Ho et al. This structure successfully controls the reflectance and transmittance through glass within the wavelength range from ultraviolet to near-infrared. The RCWA and genetic algorithm were incorporated into numerical programs to improve the spectrum properties for energy-saving structures [37]. Fernández-Hurtado et al. presented a 2D periodic pattern composed of arrays of square holes distributed inside a doped silicon substrate. This structure can be modified to increase the near-field radiative heat transfer using extended nano-structures, as shown in Figure 4c. They used the RCWA method and indicated that the near-field radiative heat conductance at room temperature, which was much greater than any nano-structured materials, can be achieved through the Si-based metasurfaces with 2D holes embedded in the periodic arrays [38]. Wang et al. applied finite-difference time domain (FDTD) simulation method to explore a 2D rectangular SiC grating structure on top of a photonic crystal (PC) and demonstrated that the TE and TM waves can excite the surface phonon polaritons (SPhPs) when they are scattered by a 2D grating structure, as shown in Figure 4d [39].

**Figure 5:**
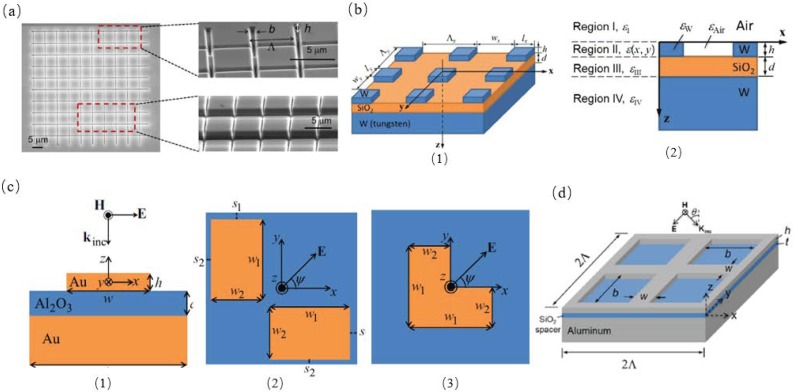
(**a**) SEM image of 2D SiC metasurfaces (pattern 1) fabricated by the focused-ion beam technique on a SiC wafer, and the close-up SEM images of the pattern edge and center areas with grating period (Λ), depth (*h*), and groove width (*b*) [40]. (**b**) Illustration of the numerical model for the 2D grating/thin-film nanostructure. The parameters used in the present study are Λ${}_{x}$ = Λ${}_{y}$ = 600 nm and *l*${}_{x}$ = *l*${}_{y}$ = 300 nm; thus *w*${}_{x}$ = *w*${}_{y}$ = 300 nm: (1) schematic of the structure; (2) side view of the structure showing different regions and the dielectric functions [41]. (**c**) Schematic of the metamaterial emitter and absorber. (1) Side view of the three-layer structure; (2) top view of the double-rectangle pattern; (3) top view of the L-shape pattern. Note that gold (Au) is issued for the metallic patterns and the ground plane, and Al${}_{2}$O${}_{3}$ is used for the dielectric film or spacer [42]. (**d**) Schematic of a periodic film-coupled concave grating meta-material [43].

Yang et al. experimentally investigated the coherent and thermal emission in SiC metasurfaces which were caused by exciting magnetic polaritons. This structure, as shown in Figure 5a, clearly showed a wavelength selectivity emission peak with a height of 0.8 and numerically stimulated the emission spectrum and plotted the electromagnetic field distribution which verified the underlying physical mechanism of magnetic polaritons [40]. Zhao et al. proposed a thermal emitter with a 2D grating/thin film nanostructure, as shown in Figure 5b, and investigated the electromagnetic field distributions and the emission spectrum with the RCWA method [41]. Sakurai et al. explored the radiative properties of metamaterials with a structure of 2D pattern on a dielectric thin film which segregated the periodic pattern structure from a metal plane. One structure is a separated double-rectangle gold pattern and another is an L-shape pattern, as shown in Figure 5c. These structures showed dual-band emissivity peaks in infrared wavelength region. The FDTD method was incorporated to investigate the electromagnetic fields at the resonant frequencies which elucidated the physical mechanisms accounting for different behaviors [42]. Wang et al. proposed a 2D metallic concave grating structure on a metal film segregated by an ultra-thin dielectric spacer, as shown in Figure 5d, and investigated the radiative properties of these metamaterials. They demonstrated the absorption peaks of that structure are at the several frequencies of 8350 cm${}^{-1}$ (with absorptance α=0.89), 9970 cm${}^{-1}$ (α=0.80), 13,740 cm${}^{-1}$ (α=0.36), 18,870 cm${}^{-1}$ (α=0.43), 21,150 cm${}^{-1}$ (α=0.23), and 23,580 cm${}^{-1}$ (α=0.36) [43].

### 2.3. Hyperbolic Metamaterials

**Figure 6:**
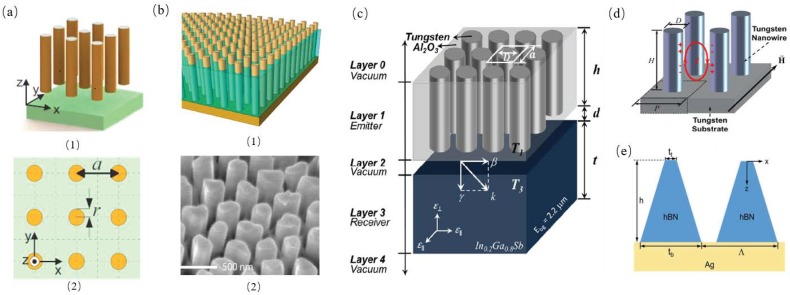
(**a**) (1) Schematic diagram (3D view and top view) of the SiC plate heat emitter (at 300 K) and the metal wire array heat absorber (at 0 K) separated by a vacuum gap. (2) Metal wires have an infinite length, radius *r* = 50 nm, and period *a* = 300 nm [45]. (**b**) (1) Schematic and (2) SEM image of partially released nickel nanowire arrays. The protruded nanowires are measured to be 400 nm from the AAO template [24]. (**c**) Schematic of a near-field TPV system consisting of a tungsten nanowire-based hyperbolic metamaterials (HMM) emitter and a TPV cell with finite thicknesses in a 5-layer structure: vacuum substrate (Layer 0), emitter (Layer 1), vacuum gap (Layer 2), receiver (Layer 3), vacuum substrate (Layer 4) [25]. (**d**)Schematic of the tungsten nanowire-based selective absorber [46]. (**e**) Schematic of the proposed hBN trapezoidal grating structure [47].

Smolyaninov et al. investigated the physical mechanism of radiative heat transfer in the hyperbolic metamaterials and predicted a huge increase of radiative heat transfer compared to the Stefan-Boltzmann law in vacuum because of the broad divergence of the photonic density of states for the first time. They demonstrated that the radiative heat transfer may approach or even exceed the thermal conduction and predicted emerging engineering applications, including thermal management [44]. The structure of a broadband thermal emitter/absorber based on the hyperbolic metamaterials is shown in Figure 6a; that was originally proposed by Liu et al. They used a numerical simulation method to investigate the heat transfer physical mechanisms of metamaterials hinged on the Wiener chaos expansion method instead of using the effective medium approximation method [45]. Shi et al. experimentally investigated the near-field thermal energy transfer enhancement in a thermal energy extraction device, as shown in Figure 6b, and found than the near-field thermal energy transfer within the thermal extraction was boosted up to around one order of magnitude with the same gap between the emitter and absorber, compared with that case without thermal extraction [24]. Chang et al. proposed a thermophotovoltaic (TPV) system consists of In${}_{0.2}$Ga${}_{0.8}$Sb and thermal emitter composed of tungsten nanowires arrays embedded inside the Al${}_{2}$O${}_{3}$ host within a nanometer vacuum gap, as shown in Figure 6c. They used the fluctuational electrodynamics combined with the effective medium theory to calculate the near-field radiative heat flux and found that the radiative energy conversion was boosted by the epsilon-near-pole and hyperbolic modes at varies polarizations [25]. Chang et al. investigated nano-wired metamaterials based selective solar absorber, as shown in Figure 6d, and employed the FDTD method to do the numerical simulation for that structured design, which showed that the spectrum selectivity was due to the excitation of magnetic polariton. They employed the inductor-capacitor circuit model to predict the resonance selective wavelength of the magnetic polariton harmonic modes. They also simulated the effects of different geometric parameters such as different nanowire diameter, height, and array period [46]. Zhao et al. theoretically showed that trapezoidal gratings made of a natural hyperbolic material on a metal substrate, as shown in Figure 6e, can be used to achieve omnidirectional perfect absorption in a relatively broad spectral region. They calculated the absorptance as well as the local power dissipation and field distributions with the anisotropic rigorous-coupled wave analysis [47].

### 2.4. Multi-Layered Structures

**Figure 7:**
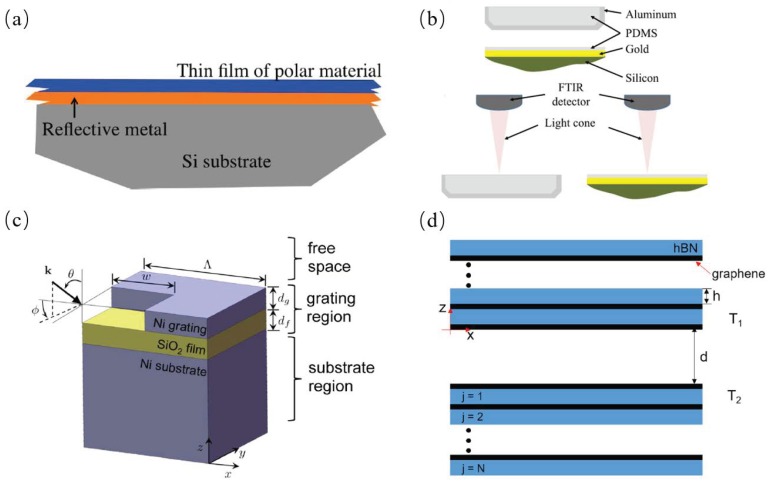
(**a**) A free-standing thin film of polar material [21]. (**b**) Geometry of bulk Polydimethylsiloxane (PDMS) samples prepared in aluminum dishes. PDMS thin film on top of gold coated silicon substrate [48]. (**c**) Schematic of one period of a 2-D Ni grating with a SiO${}_{2}$ film on a Ni substrate [49]. (**d**) Schematic of near-field radiative heat transfer between two graphene/hBN heterostructures [50].

Narayanaswamy et al. proved that very thin film of polar dielectric materials can be selective emitters in narrowband because of multiple reflections of light within multi-layered thin films; the multi-layered structure is shown in Figure 7a [21]. Srinivasan et al. demonstrated that polydimethylsiloxane (PDMS) on top of gold substrate can show selective emission properties in the 8 μm to 13 μm wavelength range, which is called atmospheric transmittance window. They showed that PDMS can act as a viable material for passive radiative cooling engineering applications. The measurement setup is shown in Figure 7b [48]. Lee et al. conducted the analysis of high temperature absorber with a structure of a 2D nickel grating atop a thin SiO${}_{2}$ film and a nickel substrate, as shown in Figure 7c, they determined the size of the 2D grating with the Taguchi method optimizing the spectral absorptance in both *S* and *P* polarizations [49]. Zhao et al. studied the radiative heat transfer physical mechanism within multi-layered structure consisting of a periodic petition of a graphene sheet and a hexagonal boron nitride (hBN) sheet, as shown in Figure 7d, and found that the near-field radiative heat transfer within this structure was not enhanced linearly according to the number of layers in that stack. This shows that it is a way to control near-field heat transfer rate through the number of graphene layers [50].

**Figure 8:**
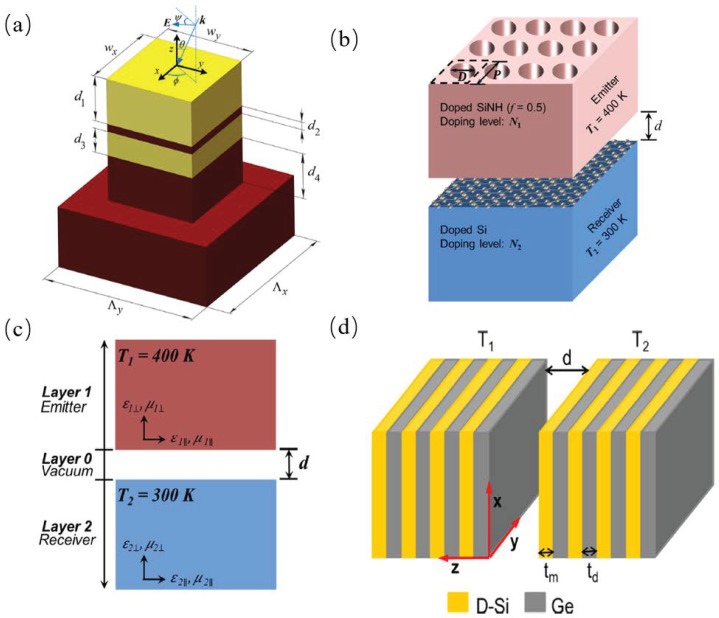
(**a**) Schematic of the proposed 2D layered grating structure made of SiO${}_{2}$ and tungsten [51]. (**b**) Schematic of the simulated structure separated by vacuum gap *d* where both the doped SiNH emitter and graphene covered D-Si receiver are assumed to be semi-infinite [54]. (**c**) Schematic of two homogeneous semi-infinite uniaxial electromagnetic metamaterials at different temperatures separated by a nanometer vacuum gap *d* [52]. (**d**) Illustration of radiative heat transfer between two multilayered metamaterials (at temperatures T${}_{1}$ and T${}_{2}$, respectively) separated by a vacuum gap of distance *d* [53].

Zhao et al. proposed a 2D grating structure of SiO${}_{2}$ and tungsten, as shown in Figure 8a, which show thermal emission control at a narrowband wavelength range and was used as a potential thermal emitter in thermophotovoltaic engineering applications [51]. Chang et al. theoretically demonstrated that enhanced near-field radiative heat transfer can be predicted within a nanostructured metamaterial thermal emitter and a graphene covered planar receiver, as shown in Figure 8b, the distance between the emitter and receiver is 20 nm. The radiative heat transfer flux was enhanced 500 times higher that the far-field radiative heat transfer between two metasurfaces, as shown in Figure 8c, using the method of fluctuational electrodynamics incorporated with anisotropic wave optics. They demonstrated that the *s*-polarized waves are more significant than the *p*-polarized waves [52]. Liu et al. proposed a multi-layered metamaterial with doped silicon and germanium separated by a vacuum gap, as shown in Figure 8d, and predicted that radiative heat transfer between multi-layered metamaterials using the fluctuational electrodynamics theory based on the Green’s formulation [53].

### 2.5. Phase Change Metamaterials

**Figure 9:**
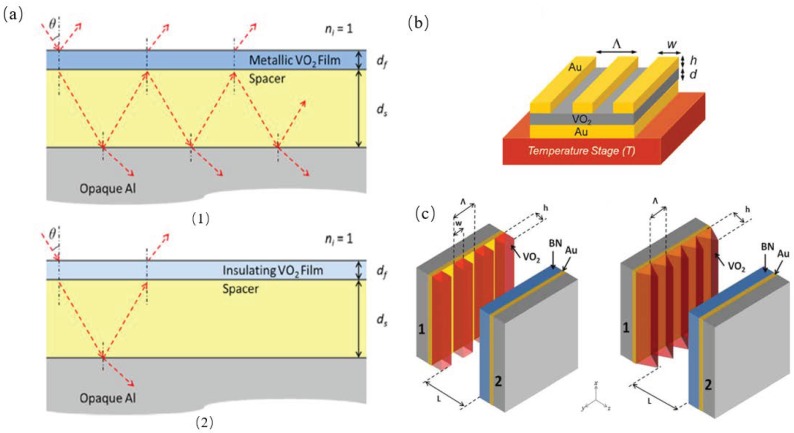
(**a**) Schematic of the proposed structure and wave propagation when the VO${}_{2}$ top layer is (1) metallic (*T* > 345 K) and (2) insulating (*T* < 341 K), where d*f* and d*s* are the film thicknesses of the VO${}_{2}$ and spacer (*n* = 3.4) respectively [55]. (**b**) Schematic of the proposed switchable metamaterial absorber/emitter made of one-dimensional gold grating (period Λ = 1 μm, stripe width *w* = 0.5 μm, and height *h* = 80 nm), a thin VO${}_{2}$ layer (thickness *d* = 80 nm), and an opaque bottom gold substrate [56]. (**c**) Schematics of near-field thermal diodes. Left: Active side has a top layer of 1-D rectangular grating made of VO${}_{2}$ of height *h*, width *w*, period Λ, and filling ratio ϕ on a gold layer deposited on a substrate. Right: Rectangular grating is replaced by a triangular one of height *h* and period Λ. Passive counterpart of both designs consists of a BN layer on gold on the top of a substrate [26].

Taylor et al. proposed an asymmetric Fabry-Perot emitter using phase change material Vanadium dioxide (VO${}_{2}$) and an opaque aluminum substrate to form an asymmetric Fabry-Perot emitter, as shown in Figure 9a. They calculated the radiative properties using uniaxial transfer matrix method and Bruggeman effective medium theory, which showed that the structure is highly reflective at the temperature below 341 K when VO${}_{2}$ is in dielectric phase. When it is above 345 K, the structure acted as a Fabry-Perot resonance cavity which has a high broadband emissivity window around 10 μm wavelength [55]. Wang et al. numerically demonstrated a switchable absorber/emitter through thermally turning on and off the magnetic resonance excitation with the phase transformation of Vanadium dioxide (VO${}_{2}$); the structure they proposed is as shown in Figure 9b. A perfect absorption peak was discovered at 5 μm when the magnetic resonance excitation occurred within the insulating VO${}_{2}$ spacer layer [56]. Ghanekar et al. showed that thermal rectification can be enhanced within the 1D rectangular and triangular VO${}_{2}$ surface gratings structure, as shown in Figure 9c. Near-field rectification ratio was boosted up dramatically because of the tunneling of surface waves from the interfaces of negative polarity. They also predicted that the rectification ratio as high as 16 occurred in a minimum temperature difference of 20 K which is the maximum in existing literature at comparable operating temperatures and gaps.

## 3. Engineering Applications

### 3.1. Thermal Absorber or Emitter for Thermophotovolatic

**Figure 10:**
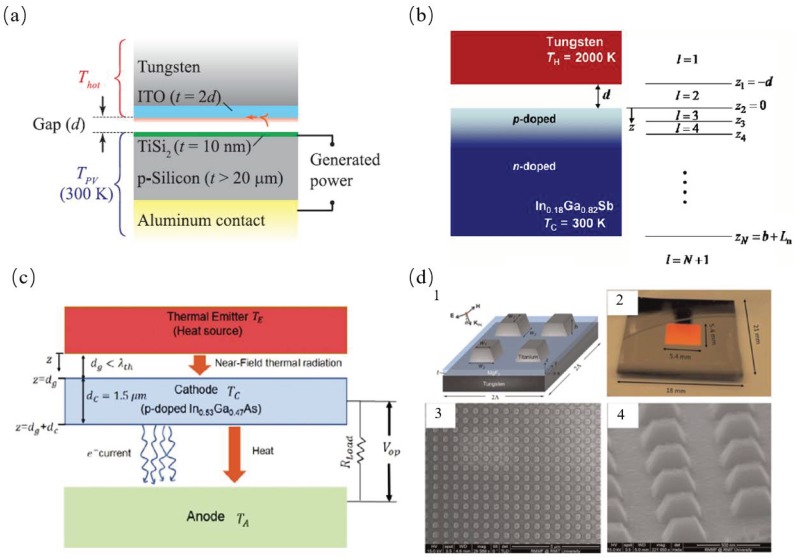
(**a**) Cross section of the hot carrier-based near-field thermophotovoltaic generator considered in this work. The indium-tin-oxide (ITO) layer supports a thermally excited surface resonance at frequency (0.61 eV) slightly superior to the barrier height (ϕ${}_{b}$) of the TiSi${}_{2}$-silicon Schottky junction (0.45 eV) [57]. (**b**) Schematic of a near-field TPV system, where In${}_{0.18}$Ga${}_{0.82}$Sb is used as the TPV cell material and plain tungsten is used as the emitter. Both the emitter and the cell material are modeled as semi-infinite media [58]. (**c**) Schematics of a NETEC system illustrating its heat transfer and carrier transport mechanisms (not scaled) [4]. (**d**) (1) Structure schematic for proposed metamaterial solar absorber. K${}_{inc}$ represents the incident wave vector, and the incidence angle θ is defined as the angle between K${}_{inc}$ and the surface normal. (2) A photo of the fabricated sample for optical characterization. SEM images of the fabricated absorber sample from (3) top view and (4) side view [5].

St-Gelais et al. showed that 10–30% conversion efficiency and 10–500 W/cm${}^{2}$ generated power densities (at 900–1500 K temperatures) can be gained after optimization of their thermal radiation spectrum with the thermal emitter in hot-carrier based near-field thermophotovoltaics (NFTPV) systems, as shown in Figure 10a. They also discussed how the near-field properties of thermal radiation were ideal fit for investigation of proposed structure for quantum efficiency hot carrier junctions [57]. Park et al. exploited local radiation absorption and photonic interaction and elucidated the radiation heat transfer in the near-field thermophotovoltaic (TPV) systems, as shown in Figure 10b. They used the fluctuation-dissipation theorem to calculate the radiation heat transfer with the multi-layered and evaluated the electric current generation using the photo-generation and recombination of electron-hole pairs in the TPV cell [4]. Ghashami et al. proposed a near-field enhanced thermionic energy conversion (NETEC) system configured with a low-bandgap semiconductor cathode separated from a thermal emitter with a subwavelength gap distance, such that a significant amount of electrons can be photo-excited by near-field thermal radiation to contribute to the enhancement of thermionic current density, as shown in Figure 10c. They also theoretically demonstrated the energy conversion efficiency of the NETEC system can exceed 40% at a significantly lower temperature than the standard thermionic generator and the near-field photoexcitation can enhance the thermionic power output by more than 10 times [58]. Wang et al. proposed and experimentally proposed a solar absorber with a structure of nanostructured titanium grating atop on an ultrathin MgF${}_{2}$ film and a tungsten ground film, as shown in Figure 10d. The normal absorptance of the solar absorber was more than 0.9 within the visible and near-infrared (IR) region but was around 0.2 in the mid-infrared (IR) region. They indicated that solar-to-heat energy conversion efficiency was 78% at 100 ${}^{\circ }$C without optical concentration structure or 80% at 400 ${}^{\circ }$C with 25 suns concentration [5]. Yang et al. used the theory of fluctuational electrodynamics to calculate the thermal rectification effect between SiO${}_{2}$ and VO${}_{2}$, which is separated by nano-sized vacuum gaps. The rectification ratio was about 1 when the vacuum gap was about 1 μm and was enhanced to 2 when the gaps became sub-20-nm gaps with emitter and receiver temperatures at 400 K and 300 K, respectively [16]. Wang et al. demonstrated thermal rectification between intrinsic Si and doped Si (rectification *R* = 2.7) and between intrinsic Si and SiO${}_{2}$ (R=9.9) can be gained at a 5 nm vacuum gap at temperatures of 1000 K and 300 K, respectively. A thermal rectifier consisting of gold and intrinsic Si with a rectification ratio about 0.85 at temperature of 600 K and 300 K when the vacuum gap is from 100nm to 500 nm [17].

**Figure 11:**
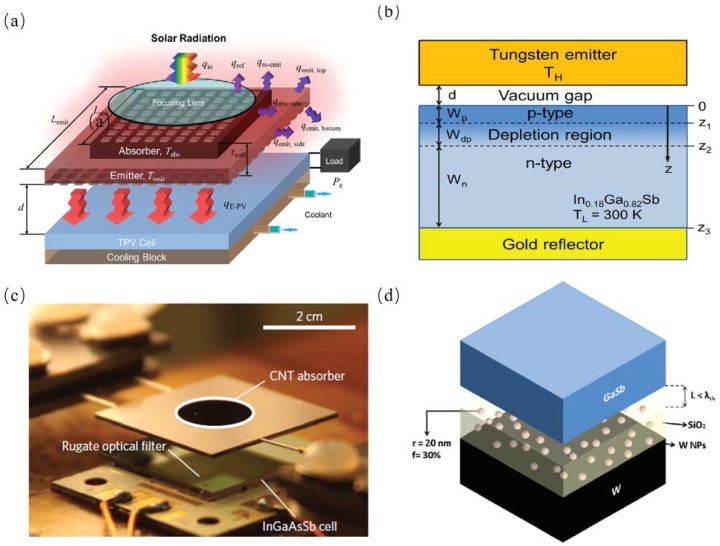
(**a**) The schematic and energy flow for an solar thermophotovoltaic (STPV) system [59]. (**b**) Schematic of the tungsten emitter and the TPV cell with different regions delineated. The In${}_{0.18}$Ga${}_{0.82}$Sb TPV cell is assumed to be at 300 K. The bottom of the active cell is either a gold mirror or a substrate with the same optical properties as the cell [22]. (**c**) Schematic of near-field thermophotovoltaic system consisting of the proposed thermal emitter and GaSb-based PV cell at separation less than the thermal wavelength [60]. (**d**) Schematic of near-field thermophotovoltaic system consisting of the proposed thermal emitter and GaSb-based PV cell at separation less than the thermal wavelength [61].

Wang et al. proposed a solar thermophotovoltaic (STPV) system with a selective absorber and emitter with an energy conversion efficiency was from 8% and 10% when the concentration factor varies from 20 and 200, as shown in Figure 11a. The energy efficiency was dramatically enhanced compared with the previous STPV system whose structure was black absorbers and emitters with an energy conversion efficiency of 2.5% [59]. Bright et al. analyzed a near-field TPV system with a gold thin film attached to the back of solar cell. They also calculated the radiative heat transfer flux between the tungsten thermal emitter at a temperature from 1250 K to 2000 K and In${}_{0.18}$Ga${}_{0.82}$Sb based TPV cell at 300K using fluctuational electrodynamics theory [22]. Bierman et al. analyzed the solar to electrical efficiency of a TPV system whose efficiency reached up to 6.8%, exceeding the performance of the photovoltaic cell alone, as shown in Figure 11b. They calculated the theoretical limits and discussed the potential engineering applications of surpassing the Shockley-Queisser limit [60]. Ghanekar et al. proposed a near-field thermophotovoltaic system consisting of a Mie-metamaterial emitter and a GaSb-based photovoltaic (PV) cell and analyzed the performance properties of this system when the separations of emitter and PV cell was less than the thermal wavelength, as shown in Figure 11d. The emitter had a SiO${}_{2}$ thin film embedded with tungsten nanoparticles with a bulk tungsten substrate. They analyzed the performance of the TPV systems at different volume fractions of tungsten nanoparticles and thickness of SiO${}_{2}$ thin film [61].

**Figure 12:**
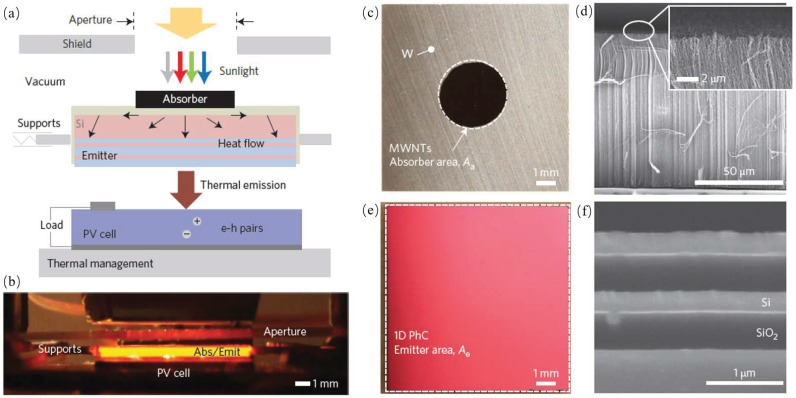
Operating principle and components of the NARO–STPV. Sunlight is converted into useful thermal emission and, ultimately, electrical power, via a hot absorber–emitter. Schematic (**a**) and optical image (**b**) of our vacuum-enclosed devices composed of an aperture/radiation-shield, an array of MWNTs as the absorber, a 1D PhC, a 0.55 eV-bandgap photovoltaic cell (InGaAsSb) and a chilled water cooling system. (**c**) Absorber-side optical image of an AR ($A_{e}$/$A_{a}$) = 10 module showing spatially defined MWNTs ($A_{a}$ = 0.1 cm${}^{2}$) on a tungsten-coated silicon substrate (1× 1 cm${}^{2}$ planar area, 550 μm thick). (**d**) SEM cross-section of the MWNTs. Inset: Magnified view of the nanotube tips. (**e**) Optical image of the 1D PhC emitter ($A_{e}$ = 1 cm${}^{2}$). (**f**) SEM cross-section of the 1D PhC showing the alternating layers of silicon and SiO${}_{2}$ [3].

Lenert et al. reported a solar thermophotovoltaic device with an experimental efficiency of 3.2%. They used a multi-walled carbon nanotube as broadband absorber and multi-layered 1 D Si/SiO${}_{2}$ photonic-crystal emitter as the absorber/emitter structure and optimized the absorber/emitter area ration to get the highest energy conversion efficiency; the structure is shown in Figure 12 [3].

### 3.2. Thermal Diode/Thermal Switch

**Figure 13:**
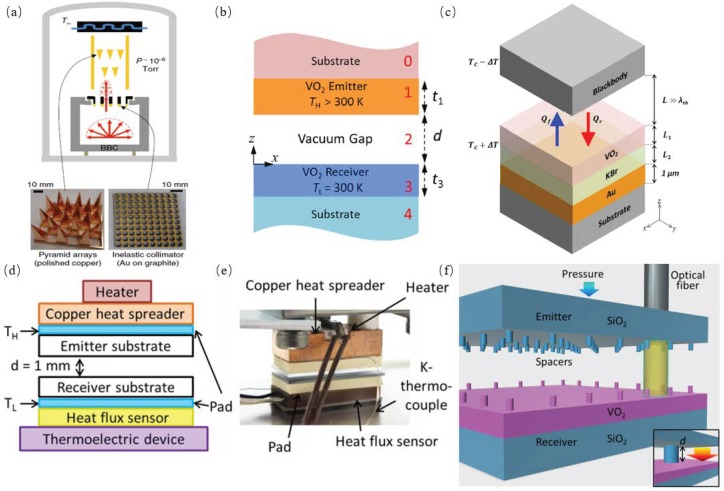
(**a**) A hot BBC (its guard heater and shields omitted for clarity) generates photons with a Lambertian distribution. The essential diode components are the inelastic thermal collimator and the test section with pyramidal mirrors. The depicted configuration is Fwd-biased, with Rev bias obtained by flipping the test section cite [10]. (**b**) Schematic of a vacuum thermal switch made of five layers with two VO${}_{2}$ thin films on substrates separated by a vacuum gap *d*. The emitter and receiver VO${}_{2}$ film thicknesses are denoted as *t*${}_{1}$ and *t*${}_{3}$, respectively. The emitter temperature is *T*${}_{H}$, which varies between 330 K and 350 K, while the receiver temperature *T*${}_{L}$ is fixed at 300 K. The directions of wave vectors are also shown [11]. (**c**) Schematic of a far-field thermal diode with a high rectification ratio. The active component has a tri-layer structure consisting of VO${}_{2}$, KBr and gold thin films on a substrate with thicknesses *L*${}_{1}$, *L*${}_{2}$ and 1 μm, respectively. The passive component is a blackbody. *T*${}_{c}$ = 341 K is the phase transition temperature of VO${}_{2}$ [64]. (**d**) Schematics of the experimental apparatus. (**e**) Photograph of the experimental apparatus. (**f**) Geometrical configuration of the experiment before the gap formation. (inset) Configuration after the gap formation.

Kats et al. experimentally investigated the infrared thermal properties of subwavelength thin film of vanadium dioxide (VO${}_{2}$) on a sapphire substrate and demonstrated that the VO${}_{2}$ thin film acted as a natural, disordered metamaterial with optical dispersion and losses in the infrared wavelength range, which will result in an absorption resonance upon the temperature tuning within the phase-transition region. They predicted engineering applications, such as radiative cooling, perfect absorber [62,63]. Chen et al. experimentally demonstrated that the ballistic mechanism required a nonlinearity so as to generate asymmetric thermal transport and that it was realized by using a thermal collimator hinged on asymmetric of the ballistic energy carrier through the pyramidal reflectors, as shown in Figure 13a. Both effects are experimentally confirmed with these pyramids and collimator. Thermal rectification of 10.9 ± 0.8% was achieved with the collimator, while almost no rectification (<0.3%) was detected without a collimator [10]. Based on the near-field thermal rectification between phase transformation materials, i.e., VO${}_{2}$, whose phase change temperature point is at 341 K, Yang et al., theoretically demonstrated a thermal switch, as shown in Figure 13b. They indicated that more than a 80% heat reduction rate was achieved at less than 30-nm vacuum gap and 50% heat reduction rate at the 1 μm vacuum gap with the fluctuational electrodynamic theory incorporated with anisotropic [11]. Ghanekar et al. proposed a far-field radiative rectification device using VO${}_{2}$ to achieve a high thermal rectification ratio in radiative heat transfer; the structure is shown in Figure 13c. Thermal rectification of more than 11 was gained at a temperature asymmetric of 20 K, which is higher than ever predicted for far-field radiative diode configurations [64]. Ito et al. experimentally investigated a radiative thermal rectifier consisting of a VO${}_{2}$ thin film on top of the silicon wafer and obtained a rectification ratio of two with 1-D steady state heat flux measurement apparatus; the experimental system was shown in Figure 13d,e. They also developed a theory model to describe the thermal rectification ratio at certain optical responses according to the measured mid-infrared reflection spectrum [65]. Ito et al. dynamically investigated by experiment the near field radiative heat transfer between SiO${}_{2}$ surface and tungsten-doped VO${}_{2}$ surface during the metal-insulator transition. They used the microfabricated spacers and applied certain pressure to keep the gap of two different surfaces at a 370 nm gap with an area of 1.6 cm${}^{2}$, the structure is shown in Figure 13f, and applied the sum of the radiative and the parasitic conductive components to model the near-field heat transfer. Their work provided a way to perform near-field thermal management dynamically without changing mechanical configuration [66].

### 3.3. Radiative Cooling

**Figure 14:**
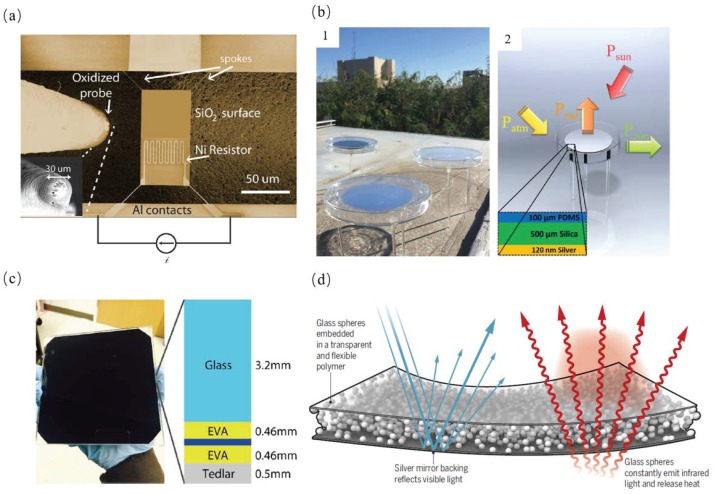
(**a**) Top view SEM image of the suspended SiO${}_{2}$ membrane with integrated resistor and the probe close to it. Inset shows zoom-in of the SiO${}_{2}$ coated probe tip [14]. (**b**) (1) Image of the samples under field test on the roof of a building. (2) Schematic of the test setup. The input/output energy balance is labeled with *P*${}_{rad}$, *P*${}_{sun}$, *P*${}_{atm}$, *P*${}_{con}$ denoting the radiated power from the cooler, absorbed power from the sun, absorbed power from the atmosphere, and conduction/convection power loss, respectively. The inset in (2) shows the cross section of the cooler structure consisting of three layers [15]. (**c**) A cross-section schematic (right) of a solar cell with encapsulation, with a 3.2 mm front glass cover, top and bottom 0.46 mm EVA film, and a 0.5 mm back sheet [67]. (**d**) A schematic of the polymer-based hybrid metamaterial with randomly distributed SiO${}_{2}$ microsphere inclusions for large-scale radiative cooling [68,69].

Guha et al. experimentally demonstrated that near-field radiative cooling can efficiently cool down hotspots by tens of degrees at several hundred nanometer gaps, as shown in Figure 14a. They showed that the measured results of radiative cooling fitted well with the theoretical predictions [14]. Kou et al. demonstrated that a polymer-coated fused silica mirror can act as a near ideal blackbody in the mid-infrared wavelength range and near ideal reflector in the visible spectrum, which can achieve radiative cooling down to below air temperature even under the sunlight (48.2 ${}^{\circ }$C) and at night (8.4 ${}^{\circ }$C), as shown in Figure 14b. It performed better than a multi-layered thin film by 3 ${}^{\circ }$C. They also estimated this cooler can achieve an average cooling power of 127 W/m${}^{2}$ during daytime [15]. Li et al. proposed a photonic radiative cooler consisting of 1D photonic films which can strongly radiate thermal emission in the infrared region while also dramatically reflected the visible and ultraviolet regimes. They showed that the solar panel can cool down by 5.7 ${}^{\circ }$C, as shown in Figure 14c and it can also be used in concentrated photovoltaic system to cool down the solar cell [67]. Zhai et al. proposed a structure of polymeric matrix randomly embedded with polar dielectric microspheres which are transparent to the visible spectrum which had an infrared emissivity more than 0.93 within the atmospheric window, as shown in Figure 14d [68,69].

## 4. Perspectives

Thermal radiation, one of the three widely known categories of heat transfer, has begun playing a significant role in thermal science and it has great engineering applications in the fields of thermal management, renewable energy harnessing, thermal biosensing, and radiative cooling. Far-field radiative thermal transport, also known as classical radiation, cannot be ignored or simply assumed to be negligible at the nano-scale.Numerous theoretical and experimental works during the past few decades have shown that both near-field and far-field radiative heat transfers in photonic metamaterials exhibit novel thermal and optical properties, such as tunable radiative wavelength selectivity, by using nano-engineered materials.

Radiative wavelength selectivity in materials is a key feature in applications such as enhanced thermal energy conversion and radiative cooling. Interdisciplinary approaches and works towards maturing these technologies will have great benefits in the fields of thermal science, novel sensing, green energy, and bioengineering. As key features of structures approach thermal wavelengths at a given temperature, a narrowband (sharp) or wideband of wavelengths can be created or shifted in emission and/or reflection spectra of nanoscale (meta-)materials. Due to near-field effects, the phenomenon of radiative wavelength selectivity becomes obvious. In addition, it can be an explorable technique for thermophotovoltaic energy harvesting, nanoscale biosensing, and energy saving by radiative cooling in the near future.
